# No evidence for neuronal damage or astrocytic activation in cerebrospinal fluid of Neuro-COVID-19 patients with long-term persistent headache

**DOI:** 10.1186/s42466-023-00277-1

**Published:** 2023-09-28

**Authors:** Laura de Boni, Alexandru Odainic, Natalie Gancarczyk, Luisa Kaluza, Christian P. Strassburg, Xenia A. K. Kersting, Ullrich Wüllner, Susanne V. Schmidt, Gabor C. Petzold

**Affiliations:** 1 Institute of Aerospace Medicine, German Aerospace Center, Cologne, Germany; 2 Division of Vascular Neurology, Department of Neurology, University Hospital Bonn, Venusberg-Campus 1, 53127 Bonn, Germany; 3 Institute of Innate Immunity, University Hospital Bonn, Bonn, Germany; 4 Institute of Clinical Chemistry and Clinical Pharmacology, University Hospital Bonn, Bonn, Germany; 5 Department of Microbiology and Immunology, The Peter Doherty Institute for Infection & Immunity, University of Melbourne, Melbourne, VIC 3010 Australia; 6 Department of Internal Medicine I, University Hospital Bonn, Bonn, Germany; 7 Department of Psychiatry and Psychotherapy, University Hospital Mainz, Mainz, Germany; 8 German Center for Neurodegenerative Diseases (DZNE), Bonn, Germany; 9 Department of Neurodegenerative Diseases and Gerontopsychiatry, University Hospital Bonn, Bonn, Germany

**Keywords:** Post-acute sequelae of SARS-CoV-2 infection, post-COVID-19, Headache, NfL, GFAP, UCH-L1, Tau, Biomarkers, Cerebrospinal fluid

## Abstract

Headache is one of the most common neurological manifestations of COVID-19, but it is unclear whether chronic headache as a symptom of Post-COVID-19 is associated with ongoing CNS damage. We compared cerebrospinal fluid (CSF) levels of markers of CNS damage and inflammation in Post-COVID-19 patients with persistent headache to hospitalized acute COVID-19 patients with neurological symptoms and to non-COVID-19 disease-controls. CSF levels of neurofilament light chain, Ubiquitin carboxyl-terminal hydrolase L1 and Tau were similar in patients with persistent headache in post-COVID-19 compared to acute COVID-19 patients and all control groups. Levels of glial fibrillary astrocytic protein were lower in patients with persistent headache in post-COVID-19 compared to some control groups of patients with neurological disease. Therefore, our pilot study of CSF markers indicates that persistent post-COVID-19 headache is not a sign of underlying neuronal damage or glial activation.

**Supplementary Information:**

The online version contains supplementary material available at 10.1186/s42466-023-00277-1.

Headache is one of the most common neurological manifestations during an acute infection with the severe acute respiratory syndrome coronavirus 2 (SARS-CoV-2), and is a leading symptom of post-acute sequelae of SARS-CoV-2 (PASC), also known as Post-COVID-19 [1]. Headache can be a primary symptom of a direct viral infection of the central nervous system (CNS), but also a secondary symptom due to autoimmune mechanisms, parenchymal hypoxia or microvascular injuries [1]. We recently reported that neuro-COVID-19 patients with long-term persisting headache showed no serological evidence for neuronal damage or reactive gliosis [2], but more direct evidence from CNS compartments such as cerebrospinal fluid (CSF) has been missing. Biomarkers for neuronal injury (neurofilament light chain (NfL), Tau) or reactive astrogliosis (glial fibrillary astrocytic protein, GFAP) were increased in patients with an acute SARS-CoV-2 infection [3–6] and higher NfL levels in CSF correlated with neurologic disability in severe COVID-19 [7] but data on persistent headache in Post-COVID-19 are scarce.

We investigated markers of neuronal injury and neuroinflammation – NfL, Ubiquitin carboxyl-terminal hydrolase L1 (UCH-L1), Tau, and GFAP – in CSF of seven COVID-19 patients with new daily persistent headache defined as being different from previous primary headaches (if any), having started after the initial serological diagnosis of SARS-CoV-2 infection and persisting longer than 3 weeks (patient characteristics are given in Table 1 and Additional File 1). Samples were taken between 3 and 37 weeks after acute SARS-CoV-2 infection (Table 1 and Additional File 1). The quality of Post-COVID-19 headaches was described as a pounding or squeezing sensation, and the intensity was described as fluctuating between medium-intensity and high-intensity. The frequency was described as daily. These patients had been classified as mild during the acute SARS-CoV-2 infection according to the WHO definition, i.e. they did not require high flow oxygen therapy or ventilation. In comparison, we also analyzed these markers in CSF from acute neuro-COVID-19 patients within the first week after the initial infection (patients of this cohort were classified as mild according to the WHO definition) and from COVID-19-negative disease-control subjects (primary headache, facial paralysis, multiple sclerosis (MS)/optic neuritis (ON), Parkinson’s disease, epileptic seizure, psychiatric disease; Table 1). Acute neuro-COVID-19 patients exhibited facial paralysis, impaired gait, seizures, or encephalopathy as their main COVID-19 symptom (Additional File 1). Samples from the SARS-CoV-2-naive control subjects were taken during clinical visits before the pandemic. Measurements of neuronal and glial markers in CSF were performed on a SIMOA analyzer (Quanterix) using the Neurology 4-Plex A (Nf-L, Tau, GFAP, UCH-L1) kit. Blood biomarkers from subgroups of patients with persistent daily headache (#1–6) and acute mild SARS-CoV-2 infection (#8–10) have been reported previously (Additional File 1) [2]. All groups were compared using Kruskal-Wallis test followed by Dunn’s multiple comparison test.

**Table 1:** Patient characteristics

	Persistent post-COVID-19 headache (n = 7)	Mild Neuro-COVID-19 (n = 5)	Control group headache (n = 5)	Control group facial paralysis (n = 5)	Control group MS/ON (n = 8)	Control group PD (n = 7)	Control group seizure (n = 5)	Control group psychiatric disease (n = 6)
Weeks since COVID-19 infection	12 ± 12	0 ± 0	n.a.	n.a.	n.a.	n.a.	n.a.	n.a.
Age [years]	40 ± 10	65 ± 8	28 ± 12	38 ± 18	42 ± 14	63 ± 13	42 ± 16	35 ± 13
Male	1 (14%)	3 (60%)	3 (60%)	4 (80%)	5 (63%)	7 (100%)	4 (80%)	6 (100%)
Female	6 (86%)	2 (40%)	2 (40%)	1 (20%)	3 (38%)	0 (0%)	1 (20%)	0 (0%)

Legend: MS = Multiple Sclerosis, OS = optic neuritis, PD = Parkinson’s disease (data are displayed as mean ± s.d.)

We found that levels of NfL, UCH-L1, Tau or GFAP were not significantly elevated in patients with persistent Post-COVID-19 headache compared to acute COVID-19 patients or all other non-COVID-19 controls (Fig. 1 A-D). Rather, GFAP levels were significantly lower in patients with persistent Post-COVID-19 headache compared to patients with MS/ON (p = 0.03) and patients with Parkinson’s disease (p < 0.0001, Fig. 1D). Mild neuro-COVID-19 patients also exhibited significantly lower GFAP levels compared to PD patients (p = 0.01, Fig. 1D). Interestingly, mild neuro-COVID-19 patients exhibited the highest Tau levels of all analyzed groups, and Tau levels were significantly increased compared to patients with psychiatric diseases without any neurological symptoms (Fig. 1B).

**Fig. 1:**
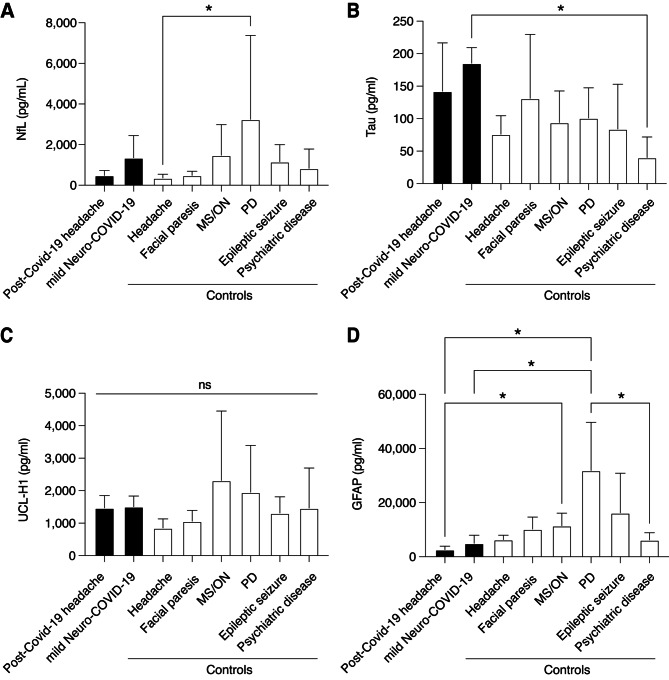
NfL, Tau, UCH-L1 and GFAP concentrations in CSF of Post-COVID-19 headache compared to acute COVID-19 and controls. (**A)** NfL levels in persistent Post-COVID-19 headache were similar compared to mild neuro-COVID-19 and COVID-19-negative control subjects (headache, facial paralysis, multiple sclerosis (MS)/optic neuritis (ON), Parkinson’s disease (PD), epileptic seizure and psychiatric disease). **(B)** Tau levels in persistent Post-COVID-19 headache were similar compared to mild neuro-COVID-19 and COVID-19-negative control subjects (headache, facial paralysis, MS/ON, PD, epileptic seizure and psychiatric disease). (**C)** UCH-L1 levels in persistent Post-COVID-19 headache were similar compared to mild neuro-COVID-19 and COVID-19-negative control subjects (headache, facial paralysis, MS/ON, PD, epileptic seizure and psychiatric disease). (**D)** GFAP levels in persistent Post-COVID-19 headache were similar compared to mild neuro-COVID-19 and some COVID-19-negative control subjects (headache, facial paralysis, epileptic seizure and psychiatric disease), but significantly lower compared to others (MS/ON, PD). All groups were compared using Kruskal-Wallis test followed by Dunn’s multiple comparison test and are displayed as mean ± s.d. (* indicates p < 0.05)

Our data suggest that persistent Post-COVID-19 headache is not an indicator of structural brain damage. These findings support recent CSF analyses demonstrating the absence of SARS-CoV-2 RNA or antibodies as a cause of neurological or neuropsychiatric post-COVID-19 syndrome [8]. Our results indicate, together with recent blood-based biomarker analysis [2, 9], that the severity of the acute COVID-19 disease course, but not the occurrence of neurological symptoms per se, determines the likelihood of neuronal injury due to SARS-CoV-2 infection itself and/or inflammatory immune reactions. In addition, our data suggest that even cases of mild neuro-COVID-19 may be associated with increased Tau CSF levels, in line with recent work on blood-based biomarkers [10]. We acknowledge the limitation that the sample size of our study was small, and that larger controlled studies will be needed to investigate these consequences of persistent headache after COVID-19 beyond biomarker signatures.

### Electronic supplementary material

Below is the link to the electronic supplementary material.

**Additional file 1: Table 1**. Detailed patient characteristics

## Data Availability

The datasets used and analyzed during the current study are available from the corresponding author on reasonable request.

## Declarations

### Trial registration

NCT05836428.
