# Supplementary material for: No evidence for neuronal damage or astrocytic activation in cerebrospinal fluid of Neuro-COVID-19 patients with long-term persistent headache
Source: Neurol Res Pract. 2023 Sep 28;5:49. doi: 10.1186/s42466-023-00277-1 (PMC10536703; doi:10.1186/s42466-023-00277-1)
Supplement: Supplementary file 1 — Additional file 1: Table 1. Detailed patient characteristics [file 42466_2023_277_MOESM1_ESM.pdf]

Additional file 1

Table 1. Detailed patient characteristics.

Group	Patient #	Primary symptom / diagnosis	Secondary diagnosis	Weeks since COVID-19 infection	Age [years]	Gender	Neurological exam	cMRI	CSF analysis / OCB
Persistent post-COVID-19 headache	1	Headache	Migraine	16	49	Female	normal	n.a.	No inflammatory change / normal
	2	Headache	Recurrent syncopes	3	23	Female	normal	normal	No inflammatory change / normal
	3	Headache	None	9	46	Female	normal	normal	No inflammatory change / normal
	4	Headache	Bechterew's disease	8	35	Female	Fasciculations right M. vastus med.	normal	No inflammatory change / normal
	5	Headache	None	37	37	Female	normal	n.a.	No inflammatory change / normal
	6	Headache	None	7	51	Female	normal	n.a.	No inflammatory change / normal
	7	Headache	None	4	37	Male	Normal	n.a.	No inflammatory change / normal
Mild Neuro-COVID-19	8	Encephalopa thy	Chronic heart disease, CAD, hypercholesterolemia, asthma	0	64	Male	Delirium, reduced vigilance, attention deficit, confusion, agitation	normal	No inflammatory change / normal
	9	Impaired gait	Hyperthyroidism, reflux	0	64	Male	Sensory ataxia	Microangiopathy	No inflammatory change / normal
	10	Encephalopa thy	Pneumonia, carotid stenosis	0	74	Female	Somnolence, increased muscle tone	normal	No inflammatory change / normal
	11	Facial paralysis	Hypertension, Diabetes Type II, hypercholesterolemia	0	54	Male	Facial paralysis	n.a.	No inflammatory change / normal
	12	Seizure	Hypothyroidism	0	71	Female	normal	Microangiopathy	No inflammatory change / normal
Disease-control headache	13	Headache	Hypertension	n.a.	43	Male	n.a.	n.a.	n.a.
	14	Headache	Von Willebrandt deficiency	n.a.	16	Male	n.a.	n.a.	n.a.
	15	Headache	None	n.a.	18	Female	n.a.	n.a.	n.a.
	16	Headache	None	n.a.	36	Male	n.a.	n.a.	n.a.
	17	Headache	None	n.a.	27	Female	n.a.	n.a.	n.a.
Disease-control facial paralysis	18	Facial paralysis	None	n.a.	50	Female	n.a.	n.a.	n.a.
	19	Facial paralysis	None	n.a.	26	Male	n.a.	n.a.	n.a.
	20	Facial paralysis	None	n.a.	16	Male	n.a.	n.a.	n.a.
	21	Facial paralysis	CAD, Hypertension	n.a.	62	Male	n.a.	n.a.	n.a.
	22	Facial paralysis	None	n.a.	36	Male	n.a.	n.a.	n.a.

Disease-control MS/ON	23	MS	None	n.a.	31	Male	Uvula deviation, saccadic gaze, reflex differences, dysdiadochokinesis	Multiple MS-typical lesions	n.a.
	24	MS	None	n.a.	42	Female	n.a.	n.a.	n.a.
	25	MS	None	n.a.	53	Female	n.a.	Multiple MS-typical lesions	n.a.
	26	MS	COPD	n.a.	64	Male	n.a.	Multiple MS-typical lesions	n.a.
	27	MS	None	n.a.	43	Male	n.a.	Multiple MS-typical lesions	n.a.
	28	MS	Lumbar disc prolapse	n.a.	52	Female	n.a.	Multiple MS-typical lesions	n.a.
	29	ON	Aortic valve defect	n.a.	22	Male	n.a.	n.a.	n.a.
	30	ON	None	n.a.	30	Male	n.a.	n.a.	n.a.
Disease-control PD	31	PD	Plasmacytoma	n.a.	59	Male	n.a.	n.a.	n.a.
	32	PD	None	n.a.	72	Male	n.a.	n.a.	n.a.
	33	PD	Polyneuropathy	n.a.	66	Male	n.a.	n.a.	n.a.
	34	PD	Gout	n.a.	68	Male	n.a.	n.a.	n.a.
	35	PD	Normal pressure hydrozephalus	n.a.	74	Male	n.a.	n.a.	n.a.
	36	PD	Polyneuropathy, cardiac carcinoma	n.a.	64	Male	n.a.	n.a.	n.a.
	37	PD	Lumbar disc prolapse, communicating hydrocephalus	n.a.	35	Male	n.a.	n.a.	n.a.
Disease-control seizure	38	Seizure	Liver dysfunction	n.a.	54	Female	n.a.	n.a.	n.a.
	39	Seizure	None	n.a.	31	Male	n.a.	n.a.	n.a.
	40	Seizure	None	n.a.	20	Male	n.a.	n.a.	n.a.
	41	Seizure	Polyneuropathy	n.a.	50	Male	n.a.	n.a.	n.a.
	42	Seizure	CAD, heart attack, hypertension, hyper-cholesterol-emia	n.a.	55	Male	n.a.	n.a.	n.a.
Disease-control psychiatric disease	43	Psychiatric disease	Liver dysfunction	n.a.	53	Male	n.a.	n.a.	n.a.
	44	Psychiatric disease	None	n.a.	31	Male	n.a.	n.a.	n.a.
	45	Psychiatric disease	None	n.a.	27	Male	n.a.	n.a.	n.a.
	46	Psychiatric disease	Polyneuropathy	n.a.	17	Male	n.a.	n.a.	n.a.
	47	Psychiatric disease	CAD, heart attack, hypertension, hyper-cholesterol-emia	n.a.	43	Male	n.a.	n.a.	n.a.
	48	Psychiatric disease	None	n.a.	38	Male	n.a.	n.a.	n.a.

Legend: MRI, magnet resonance imaging. CSF, cerebrospinal fluid. OCB = oligoclonal bands, CAD = coronary artery disease. MS = Multiple Sclerosis, OS = optic neuritis
